# Severe Hyperammonemia Due to Fecal Bowel Obstruction With a Congenital Portosystemic Shunt Resulting in Refractory Status Epilepticus and Cerebral Edema

**DOI:** 10.7759/cureus.42452

**Published:** 2023-07-25

**Authors:** Tetsuro Kawakami, Kiyomitsu Fukaguchi, Naoko Isogai, Hiroshi Koyama

**Affiliations:** 1 Department of Pediatrics, Tokyo Metropolitan Children's Medical Center, Tokyo, JPN; 2 Division of Critical Care, Shonan Kamakura General Hospital, Kanagawa, JPN; 3 Department of Surgery, Shonan Kamakura General Hospital, Kanagawa, JPN

**Keywords:** metabolic encephalopathy, refractory status epilepticus., congenital portosystemic shunt, fecal bowel obstruction, hyperammonemia

## Abstract

Hyperammonemic encephalopathy is a neurological emergency that can lead to seizures and cerebral edema. Although early interventions have been suggested, no clear criteria have been established. Herein, we report a case of severe non-hepatic hyperammonemia resulting in refractory status epilepticus within a day. A 79-year-old woman presented with acute altered mental status. Initial evaluation revealed septic shock and hyperammonemia due to fecal bowel obstruction with congenital portosystemic shunt. The patient was unresponsive to medical treatment and developed refractory status epilepticus. After surgical drainage with colostomy and a decrease in ammonia level, the patient developed cerebral edema and did not recover from the coma. Severe hyperammonemia warrants early intervention, especially in critically ill patients, with treatment of the cause and augmented removal of ammonia with renal replacement therapy.

## Introduction

Hyperammonemic encephalopathy is a critical neurological condition characterized by seizures, cerebral edema with increased intracranial pressure, coma, and death [1-4]. In critically ill patients, hyperammonemia is commonly associated with liver disease, whereas non-hepatic causes account for less than 5% of cases [1]. Despite suggestions for early interventions, there is currently no established guideline for determining the specific threshold to initiate empiric therapy in critically ill adults with undifferentiated non-hepatic hyperammonemia (NHHA) [2]. While the duration of coma exceeding three days in pediatric patients with urea cycle disorders is known to be linked to cerebral edema and mortality, the duration leading to irreversible neurological damage in critically ill adults remains unclear [5].

In this report, we present a rare case of NHHA caused by fecal bowel obstruction in a patient with a congenital portosystemic shunt (CPSS), accompanied by septic shock and respiratory failure. The patient developed refractory status epilepticus only 17 hours after admission. Initially, the NHHA remained undifferentiated and did not respond to standard medical management. However, upon suspecting the bowel obstruction as a potential source of ammonia, surgical intervention led to a rapid decrease in ammonia levels. Unfortunately, the patient did not regain consciousness from the coma. Our objective is to share the rapid clinical progression of this case and emphasize the importance of preventing irreversible neurological damage in critically ill patients with NHHA.

## Case presentation

A 79-year-old woman was brought to the emergency department due to altered mental status. The night before the onset, according to her daughter, the patient had abdominal pain after dinner, vomited a few times, and then both pain and vomiting resolved. In the morning, the patient had no pain and looked as usual. When her daughter went to call the patient for lunch at noon, she found the patient unresponsive on the bed and called emergency medical services. The patient had a history of constipation, with defecation occurring only two to three times per week. According to the daughter, the patient had abdominal pain and vomiting about once a month for several years, which got better in a day, and she had no trouble with her daily activities. Five years ago, during a routine health checkup, a transient hepatic dysfunction was found, and an abdominal ultrasound scan showed a portal vein shunt. However, her laboratory values spontaneously normalized and she had no hyperammonemia; therefore, she had been followed up without treatment.

On arrival, the patient was hemodynamically unstable, and vital signs were noted as follows: blood pressure of 88/63 mmHg; heart rate of 100 beats/minute, respiratory rate of 35 breaths/minute, and oxygen saturation of 92% on 10 L of oxygen with a non-rebreather mask. The initial Glasgow Coma Scale (GCS) score was 9, which deteriorated to 3 in 2 hours.　Physical examination revealed Kussmaul breathing, skin mottling was observed on the extremities and trunk, and the patient was in apparent shock. The lower abdomen was distended but soft, and tympanic to percussion. Bedside echocardiography revealed normal cardiac contraction, no pericardial fluid or right ventricular overload findings, and no venous thrombus in the lower extremities.

Laboratory studies showed hyperammonemia (630.1 μmol/L) and anion gap metabolic acidosis (pH 7.18) due to lactic acidosis (10.19 mmol/L). Urine drug screen results were negative, and the remaining test results, including liver function tests, were unremarkable (Table 1).

**Table 1:** Initial laboratory values with reference range PT, prothrombin time; APTT, activated partial thromboplastin time; AST, aspartate aminotransferase; ALT, alanine aminotransferase; LDH, lactate dehydrogenase; GTP, guanosine triphosphate; ALP, alanine phosphatase; BUN, blood urea nitrogen; Hs-TnI, high-sensitive troponin-I; CRP, C-reactive protein; pH, power of hydrogen; PaCO_2_, partial pressure of carbon dioxide

Variables	Value	Normal Range
White blood cell count	11,500	3,000–9,700/μL
Hemoglobin	16.1	11.0–15.6 mg/dL
Platelets	14.3×10^4^	12.4–30.5/μL
PT	74	77.8–130.0 %
APTT	31.6	23.6–38.0 seconds
Total bilirubin	1.1	0.1–1.2 mg/dL
Albumin	3.7	3.8–5.2 g/dL
AST	49	12–35 U/L
ALT	24	6–40 U/L
LDH	14	0–48 U/L
GTP	14	0–48 U/L
ALP	191	115–359 U/L
BUN	44.3	7.4–19.5 mg/dL
Creatinine	1.45	0.4–1.0 mg/dL
Sodium	142	135–147 mEq/L
Potassium	4.6	3.4–4.8 mEq/L
Glucose	137	70–110 mg/dL
Hs-TnI	83.82	0–18.4 pg/mL
CRP	9.8	0.0–0.5 mg/dL
Procalcitonin	>100	0.0–0.5 mg/dL
Ammonia	630.1	11.2–31.7 μmol/L
pH	7.17	7.35–7.45
PaCO_2_	51.2	41–51 mmHg
Bicarbonate	18.6	22–26 mmol/L
Base excess	-10.2	0±2 mmol/L
Lactic acid	10.19	0.5–2.2 mmol/L

**Figure 1:**
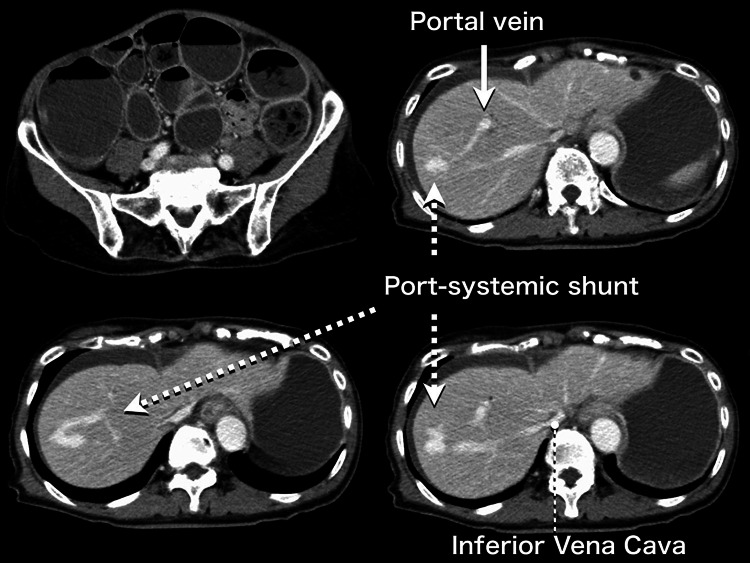
Fecal bowel obstruction and congenital portosystemic shunt Computed tomography with contrast showed a portosystemic shunt (dotted arrow) between the right hepatic vein and portal vein (solid arrow), as well as bowel obstruction by feces filled up from the rectum to the descending colon, with gastrointestinal tract distention up to the stomach without any luminal filling deficit.

Chest-to-pelvic contrast-enhanced computed tomography (CT) (Figure 1) showed a portosystemic shunt between the portal and right hepatic veins, as well as bowel obstruction by feces filled up from the rectum to the descending colon, with gastrointestinal tract distention up to the stomach without any ischemic findings such as luminal filling deficits. Plain head CT and magnetic resonance imaging findings were unremarkable, and cerebrospinal fluid analysis revealed no signs of meningitis or encephalitis. Blood cultures taken on admission revealed extended-spectrum β-lactamase-producing *Escherichia coli*. 

The patient was intubated and treated as a case of septic shock with empiric broad-spectrum antibiotics, intravenous fluids, vasopressors, and stress-dose hydrocortisone. The patient was on lactulose, rifaximin, and carnitine. After initial management, she stabilized transiently, and her serum lactate level decreased to 5.17 μmol/L.

In 17 hours after arrival, she developed refractory status epilepticus, which needed multiple anticonvulsants with increased dose of vasoactive agents. As we suspected hyperammonemic encephalopathy due to fecal bowel obstruction with CPSS as the most likely cause of the coma, we attempted bowel contents drainage by nasogastric tube and colonoscopy for bowel contents drainage. Endoscopic drainage was completed 25 hours after arrival, and the serum ammonia level remained high (513.7 μmol/L). We performed surgical drainage with colostomy, the ammonia level rapidly decreased to 99.8 μmol/L, and the requirement for vasoactive agents decreased in 29 hours after arrival.

**Figure 2:**
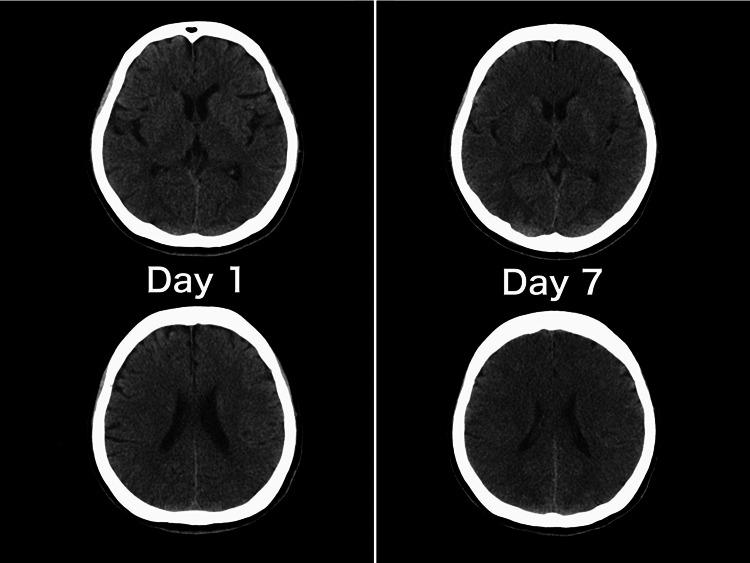
Computed tomography of the brain at day 1 and day 7. Plain head computed tomography on the first day showed no abnormalities. Findings such as narrowing of the cerebral sulci and bilateral ventricles with loss of gray-white differentiation in the temporal and frontal areas and relatively low parenchymal density suggest cerebral edema on day 7.

Intraoperative findings revealed no stenosis or mechanical obstruction, but necrosis of the superficial layer of the colonic mucosa, without perforation or total necrosis, was observed. Even after the ammonia level decreased, the GCS score did not improve, and the plain CT on day 7 (Figure 2) showed cerebral edema.

Electroencephalography performed on days 3, 7, and 14 suggested general brain dysfunction, with no epileptic findings. The coma did not improve, and the patient underwent a tracheostomy and was transferred to a nursing hospital on day 33 of hospitalization.

## Discussion

In this case, severe hyperammonemic encephalopathy due to fecal bowel obstruction with CPSS lead to coma in 2-3 hours of onset, refractory status epilepticus in 17 hours, and cerebral edema. Although the septic shock and aspiration pneumonia may also have affected cerebral edema, a timely decision on management of hyperammonemic encephalopathy could have reduced the neurological injury in this patient. Below, we discuss the pathogenesis of hyperammonemia in this case, as well as the proposed thresholds for interventions.

Adult hyperammonemia is most often the result of liver dysfunction [1-4]. The etiologies of NHHA can be broadly grouped into escalated production and impaired clearance. Production of ammonia is increased in muscle catabolism, protein load, generalized tonic-clonic seizure, intensive exercise, and a bacterial overgrowth in the gut. Infections with urease-producing bacteria such as *E. coli*, *Proteus mirabilis*, and *Ureaplasma* can also induce hyperammonemia. Clearance of ammonia can be impaired due to defects in urea production such as urea cycle disorders, and drugs inhibit the urea metabolic pathway [2]. Anatomic bypass of the liver with any portosystemic shunt also decreases clearance of ammonia [6]. In this patient, fecal bowel obstruction and *E. coli *overgrowth may have promoted intestinal ammonia generation, and bypass flow via CPSS reduced ammonia clearance [7].

A limitation of this report is the difficulty in identifying a single cause of hyperammonemia. This is because this case was complicated by sepsis, pneumonia, and renal and hepatic dysfunction. However, the patient's hyperammonemia was refractory to non-surgical therapy. The patient rapidly improved after surgery for intestinal obstruction. In addition, as ammonia levels decreased, the demand for anticonvulsants and vasoactive drugs decreased. The clinical course suggested that the neurological deficit in this case was related to persistent hyperammonemia, primarily due to CPSS and fecal bowel obstruction.

At high levels, ammonia can cross the blood-brain barrier, where astrocytic glutamine synthetase converts ammonia and glutamate to glutamine, which, in turn, acts as an osmolyte and increases cerebral volume [3]. Cerebral edema may occur along with a rapid rise in ammonia levels, particularly in patients without prior liver failure who are more susceptible to cerebral edema and death [4]. The severity of neurological injury is associated with ammonia level and duration of hyperammonemic coma [3,4,8]. In patients with acute hepatic failure, levels >100 μmol/L are predictive of hepatic encephalopathy [9], with levels >144 μmol/L increasing the risk of intracranial hypertension [10], with intracranial hypertension developing in 55% of patients with an ammonia level > 200 μmol/L [8]. In pediatric urea cycle abnormalities, high peak ammonia levels >200 μmol/L and coma sustained for three days or longer are considered high-risk factors for cerebral edema [5]. However, the threshold of blood ammonia levels and the duration of hyperammonemia for the development of cerebral edema remain uncertain in adult patients. In our case, the peak serum ammonia level was 630.1 μmol/L. Although we successfully reduced the serum ammonia level with surgical intervention, irreversible brain damage had already occurred within 30 hours of onset. To make an earlier decision regarding invasive management, hyperammonemia should be considered a neurological emergency and should have its own criteria apart from the intervention for its cause.

Interventions to hyperammonemia should be selected based on the suspected pathophysiology, but no definitive guideline exists for a specific threshold to institute empiric therapy in critically ill adults with undifferentiated NHHA. As an approach to undifferentiated NHHA in intensive care units, Long and Coursin recommend close follow-up of ammonia levels between 60 and 100 μmol/L and to initiate diagnostic evaluation and treatment to suspected cause [2]. For levels > 100 μmol/L, therapies directed at inborn errors of metabolism should be considered with consultation with a genetics team [2]. For levels >150 μmol/L, Long and Coursin recommend early initiation of exogenous ammonia clearance therapy (e.g., renal replacement therapy) while other diagnostic and therapeutic measures are deployed [2,11]. In this case, unstable hemodynamic state and respiratory failure may have affected cerebral edema, yet the emphasis is still on intervention to the severe hyperammonemia itself to prevent irreversible neurological injury.

## Conclusions

Acute altered mental status with severe hyperammonemia is a neurological emergency with imminent cerebral edema that requires timely intervention to eliminate the source of ammonia and reduce its levels. Although there has been no definitive threshold to initiate renal replacement therapy, early intervention for levels > 150 μmol/L should be considered, especially in critically ill patients.
